# Tackling type 2 diabetes in Qatar: integrating nutrition, physical activity, and policy reforms for national health advancement

**DOI:** 10.3389/fpubh.2026.1762653

**Published:** 2026-05-21

**Authors:** Ali Al-Kuwari, Amna Ali, Abdulla Al-Mutawa, Abdulla Al-Khuzaei, Christine Gaskell

**Affiliations:** Weill Cornell Medicine–Qatar, Qatar Foundation, Education City, Doha, Qatar

**Keywords:** climate-adaptive strategies, culturally tailored interventions, diabetes prevention, Gulf Cooperation Council, nutrition, physical activity, public health policy, Qatar

## Abstract

Type 2 diabetes mellitus (T2DM) is a rising public health concern in Qatar. The relationship between lifestyle factors and T2DM represents one of the most significant public health challenges facing modern societies, with particular relevance to the State of Qatar and the broader Gulf Cooperation Council (GCC) region. The Middle East and North Africa (MENA) region currently exhibits some of the highest T2DM prevalence rates globally, with the Gulf states experiencing particularly rapid increases that parallel their accelerated economic development and societal transformation. This increase in T2DM has been brought about by sedentary lifestyle-related obesity and dietary patterns rich in carbohydrates, particularly from traditional and modern convenience foods. Physical inactivity is further lowered by environmental barriers, including extreme heat, and increased screen time, adding to risks of developing T2DM. This paper highlights these lifestyle factors and proposes interventions such as inclusion of low-glycemic, fiber-dense foods, customized exercise interventions, and food labeling policies. Tackling these issues through culturally and environmentally tailored strategies is essential to reducing T2DM prevalence while preserving Qatar’s traditions. This review synthesizes evidence to propose Qatar-specific, climate-adaptive, and culturally aligned strategies not previously emphasized in the T2DM literature, offering a novel framework for future public health interventions.

## 1 Introduction - type 2 diabetes

According to the International Diabetes Federation (IDF), approximately 589 million individuals were affected by type 2 diabetes in 2024, a figure projected to increase to 853 million by 2050 (1). Furthermore, based on mathematical modeling analysis and Qatar’s fast socioeconomic transition, modernization, and dramatic lifestyle changes, the prevalence of type 2 diabetes is expected to rise locally from 7.0 to 14.0% between 2021 and 2050, one of the highest incidence and prevelance rates globally (2).

Beyond prevalence estimates, it is important to consider the substantial clinical, economic, and health system implications associated with the growing burden of T2DM in Qatar. T2DM is a leading contributor to cardiovascular disease, chronic kidney disease, neuropathy, and visual impairment, conditions that place sustained pressure on healthcare infrastructure and long-term care services (3). In a population experiencing demographic shifts and high baseline metabolic risk, these complications threaten to increase healthcare expenditures, reduce workforce productivity, and challenge the long-term sustainability of health systems (4). Addressing T2DM therefore represents not only a clinical priority but also a broader public health and socioeconomic imperative aligned with Qatar National Vision 2030 and national noncommunicable disease strategies.

As a rapidly developing nation experiencing dramatic socioeconomic transformation, Qatar presents a unique epidemiological landscape where traditional lifestyle patterns have been substantially altered by modernization, urbanization, and technological advancement, trends that are remarkably consistent across neighboring Gulf states including the United Arab Emirates, Saudi Arabia, Kuwait, Bahrain, and Oman. These shared characteristics suggest that effective interventions developed and tested in Qatar may have significant transferability and relevance for neighboring countries facing similar public health challenges.

Although several reviews have examined lifestyle-related determinants of T2DM across the Gulf Cooperation Council (GCC) and Middle East and North Africa (MENA) regions, significant gaps remain. Existing literature frequently discusses dietary transitions and physical inactivity in broad regional terms, paying limited attention to the specific environmental constraints imposed by extreme heat and humidity, the persistence of culturally embedded dietary practices, and the role of national policy mechanisms such as food subsidies, food labeling regulations, and urban planning. Furthermore, many reviews consider nutrition, physical activity, and policy interventions in isolation rather than within an integrated framework tailored to specific national contexts. As a result, there is a lack of comprehensive, context-specific models that capture how climate, culture, and policy intersect to shape T2DM risk and prevention opportunities in Qatar.

The alarming rise of T2DM diagnoses can be ascribed to factors such as sedentary lifestyles, poor dietary patterns, and insufficient physical activity. The Qatari population exhibits a high prevalence of obesity characterized by a BMI ≥ 30, with nearly 30% of individuals affected and rates reaching the mid-high 40% range among students a significant increase from previous decades (2, 5). Although the majority of cases are related to adults and older adults, the rise in youth obesity and T2DM is considerably alarming as well. In 2020, the incidence of type 2 diabetes among Qatari youth was reported at 2.51 per 100,000 (6). While T2DM traditionally affects older adults, the emergence of youth-onset diabetes presents an increasingly urgent concern. Among Qatari youth, T2DM incidence rose from 1.82 per 100,000 in 2012 to 2.51 per 100,000 in 2020, with intermediate data showing rates of 2.7 per 100,000 in 2016 (6).

Qatar’s evolving dietary landscape significantly contributes to T2DM risk factors. The traditional Qatari diet, inherently high in carbohydrates, sugar, and sodium, has been supplemented by modern eating patterns characterized by frequent fast-food consumption, ultra-processed meal dependency, and reliance on convenient delivery platforms (7). Government food security policies, while well-intentioned, have inadvertently contributed to the problem through subsidies for essential commodities including oil, sugar, and rice, potentially facilitating excessive caloric intake. The nation’s extreme climate conditions, with summer temperatures frequently exceeding 45 °C (113 °F) and high humidity levels, naturally discourage outdoor physical activities for extended periods each year, further promoting sedentary behaviors (7, 8).

In response to these gaps, this narrative review synthesizes current evidence on diet, physical activity, environmental constraints, and policy-level drivers of T2DM in Qatar to propose an integrated, climate-adaptive, and culturally aligned framework for prevention and risk reduction. Unlike prior regional reviews, this framework explicitly situates lifestyle interventions within Qatar’s extreme climatic conditions, sociocultural dietary practices, and existing public health and policy landscape. By integrating evidence across individual, community, and policy domains, this review aims to inform future national strategies, guide context-appropriate public health interventions, and offer a conceptual model that may be adaptable to other GCC countries facing similar challenges.

## 2 Methods

A comprehensive search was done using electronic databases PubMed, Scopus, and Google Scholar between the dates of January 2010 and March 2025. Keywords and Boolean operators were utilized to narrow the search to appropriate findings and were as follows:

“Type 2 diabetes” AND “Qatar” OR “Middle East” AND (“nutrition” OR “diet”) AND (“physical activity” OR “exercise”) AND (“policy” OR “public health strategy”).

Articles were included if they:

- Reported on human subjects.
- Focused on T2DM prevalence, risk factors, lifestyle determinants, or policy interventions.
- Provided data relevant to Qatar or comparable Gulf countries (GCC).

Official government/public health reports (for example, the Qatar National Physical Activity Guidelines), systematic reviews, and peer-reviewed original research articles were all included within the scope of research for a holistic perspective to be achieved. The references of the listed papers were additionally manually examined.

To enhance transparency, the narrative synthesis followed a structured workflow. Following database searches and article selection based on predefined inclusion criteria, relevant studies and reports were reviewed and organized thematically into four key domains: nutrition, physical activity, environmental constraints, and policy-level determinants. Evidence within each domain was narratively synthesized with an emphasis on relevance to the Qatari context. Insights from these thematic areas were then integrated to inform the development of the proposed conceptual framework linking lifestyle, environmental, and policy factors influencing T2DM risk in Qatar.

**Figure 1:**
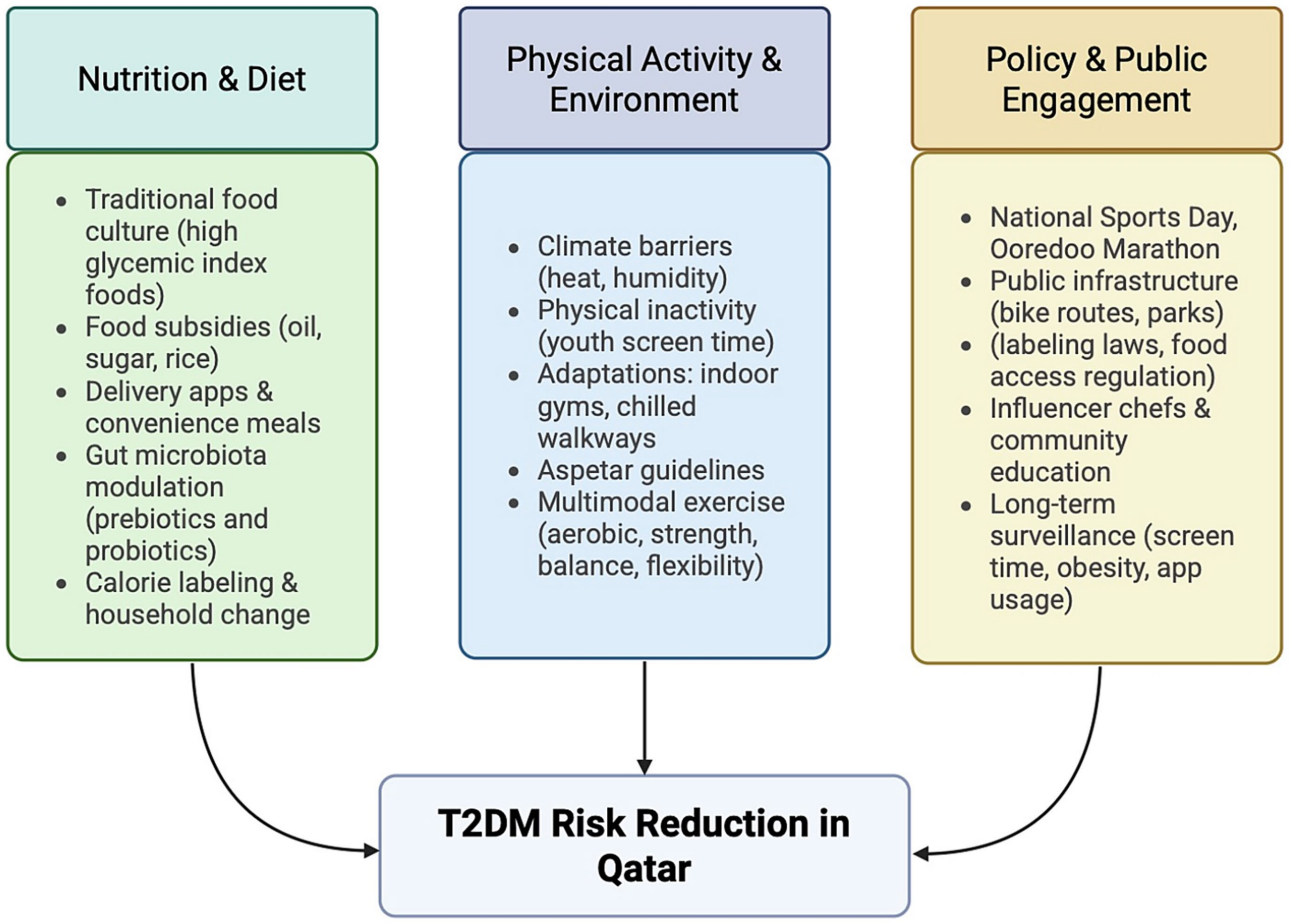
Integrated lifestyle–environment–policy framework for T2DM prevention in Qatar: This figure presents a conceptual framework illustrating how nutrition and dietary practices, physical activity and environmental constraints, and policy and public engagement interact to influence type 2 diabetes mellitus (T2DM) risk in Qatar. The three domains are interconnected and collectively contribute to T2DM risk reduction through multi-sectoral action. Arrows indicate the bidirectional relationships between lifestyle behaviors, environmental conditions, and policy-level interventions. The framework reflects the climate-adaptive and culturally aligned approach proposed in this review. Created with BioRender.com.

53 articles were selected based on the criteria listed above. The results were categorized thematically into three main areas: public health policy, physical activity and exercise, and nutritional considerations (see Figure 1). Studies from comparable GCC countries were included where Qatar-specific data were limited, given shared sociocultural, environmental, and lifestyle characteristics across the region.

## 3 Results

### 3.1 Study selection and characteristics

After database screening and eligibility evaluation based on predetermined inclusion criteria, a total of 53 studies were included in this study. Observational studies, systematic reviews, and official reports on T2DM prevalence, lifestyle-related risk factors, and public health interventions in Qatar and similar GCC populations constitute the included studies. These studies were combined conceptually into important areas such as policy-level determinants, physical activity, nutrition, and epidemiology.

### 3.2 Lifestyle-related risk factors in Qatar

Lifestyle plays a critical role in an individual’s health. A major factor that influences T2DM diabetes prevalence is the lifestyle choices and habits common in Qatar, such as regular intake of highly-processed fast foods that are heavy in calories, a lack of physical activity, excessive screen time (especially among the youth), and inconsistent sleep patterns (9). A study by Awad et al. (2) shows that there is a 7% annual prevalence of T2DM in adults in Qatar of the age ranging from 20 to 79. Moreover, the study illustrates that there are many lifestyle factors that contribute to the increase of T2DM and one of these factors is obesity; the prevalence of T2DM among obese individuals in Qatar has risen from 21.9 to 29.9% (2). Increased screen time (through use of video games and social media, among other technology engagement) has led to reduced physical activity among adolescents, contributing to higher obesity rates, consequently increasing T2DM in Qatar (7–9). We have addressed lifestyle under two main categories in relation to T2DM: Nutrition and Physical activity.

#### 3.2.1 Nutrition and dietary patterns

The nutritional landscape in Qatar has undergone a profound transformation over recent decades, characterized by a shift from traditional dietary patterns rich in whole grains, legumes, vegetables, and lean proteins toward a Western-style diet dominated by highly processed foods, refined carbohydrates, saturated fats, and energy-dense, nutrient-poor options. The dietary habits of the modern Qatari population are heavily influenced by high-carbohydrate traditional foods such as machboos (spiced rice with either meat or chicken), thareed (a bread-soaked meat and vegetable stew), and harees (a wheat and meat porridge), all of which are rich in starch and often served in large portions. Recent research has found that the mean intake of sugar amongst Qatari households is 153 g per day (10). This is significantly higher than certain countries known for healthier lifestyles and greater physical activity such as Japan and Italy which have significantly lower average sugar intakes, ranging from approximately 50 g to 70 g per day (11–13). It has also found that the typical Qatari diet is rich in meat, sugar, and sodium, while being deficient in essential nutrients such as calcium and fiber (14, 15).

Food purchased by Qatari households often exceeds the recommended energy and protein requirements guidelines set by national dietary standards, with nearly double the estimated daily intake compared to non-Qatari households; for example, Qatari households had an average daily energy (kcal) per capita of 4,275 kcal, about twice as much as non-Qatari households (2,424 kcal) (16, 17). A significant contributor to this is the food subsidy program called “Tamween,” which makes products like sugar, rice, and oil more accessible to Qatari nationals (18). The average daily energy intake provided by subsidized food items alone accounts for 41% of total daily energy intake in Qatari households, amounting to 1,753 kcal per capita per day (16, 17). While these figures represent food purchases rather than actual food consumption, the constant availability of food can lead to excessive intake which contributes to unhealthy eating patterns.

This reliance on high-carbohydrate and energy-dense foods can cause glucose spikes, increasing the risk of insulin resistance. However, neither the traditional Qatari cuisine nor the nutritional value of the food that households buy can be held exclusively responsible for the increased incidence of T2DM in Qatar. As local food delivery applications like Snoonu and Talabat provide users with convenience and a variety of options, collectively offering around 6,500 vendors, their increasing popularity may be a contributing factor to the rise in T2DM incidence. For example, during the third quarter of 2024, Talabat had between 80,000 and 86,000 weekly active users in Qatar, and Snoonu had between 52,000 and 55,000 weekly active users within the same time (19). To put that into perspective, these figures collectively represent approximately 4.7% of Qatar’s total population of 3 million. The proliferation of fast-food consumption represents a particularly concerning trend, as these foods are typically characterized by high caloric density, excessive sodium content, trans fatty acids, refined sugars, and minimal fiber content. The frequent consumption of such foods contributes to postprandial hyperglycemia, insulin resistance, weight gain, and the development of metabolic syndrome, a cluster of conditions that significantly elevate the risk of T2DM. Furthermore, the convenience culture associated with fast food consumption often displaces traditional meal preparation practices that historically emphasized fresh, locally sourced ingredients and balanced macronutrient profiles, especially in countries of the GCC (20). Research has indicated that increasing availability of ultra-processed foods is linked to increased incidence of type 2 diabetes, insulin resistance, and obesity (21). Such apps also offer inciting deals that attract users, which creates a cycle of habitual ordering, leading many to consume restaurant meals rather than freshly prepared home cooked meals (22). This means users have little to no control over nutrient intake, making it difficult to manage calorie content and portions. Although limited research exists on the long-term impact of food delivery apps on the Qatari population, further research is needed to understand their role in shaping dietary habits as they may amplify pre-existing public health issues related to diet and lifestyle.

While nutrition remains the cornerstone of diabetes prevention, its impact is magnified or mitigated by the level of physical activity, a factor strongly influenced by Qatar’s environmental constraints. Furthermore, despite these findings, there remains limited longitudinal and interventional evidence assessing the direct impact of dietary patterns on T2DM outcomes in the Qatari population.

#### 3.2.2 Physical activity and environmental constraints

Recognizing physical activity’s crucial role in disease prevention and management, a team of doctors at Aspetar hospital have established the Qatar National Physical Activity Guidelines. These guidelines serve as a thorough, evidence-based resource designed to encourage physical activity as a potent tool for preventing, controlling, and treating various chronic health diseases; these include some of the most common chronic health diseases of the country such as diabetes, heart disease, and arthritis (23). The epidemiological significance of physical inactivity in T2DM development cannot be overlooked. In Qatar, several factors have contributed to low physical activity levels across all age groups. The extreme climatic conditions, characterized by prolonged periods of intense heat and humidity, have historically limited outdoor physical activities during much of the year. However, this challenge has been compounded by rapid urbanization, which has created built environments that are not conducive to active transportation or recreational physical activity.

The Qatari government has made significant strides in facilitating and promoting a healthier and more active population, as supported by recent public health reports and policy analyses. Documented efforts include the development of outdoor walkways, public parks, gyms, and the facilitation of sports-focused events (24). The official recognition and mass-media promotion of sporting events such as National Sports Day and the annual Ooredoo Marathon, along with a growing number of privately run sporting initiatives, have been shown to enhance public engagement in physical activity (25). The literature on national health promotion strategies emphasizes how crucial these programs are for raising awareness and involvement at the community level (26). As part of its larger plan to promote a health-conscious society and discourage inactive lives, Qatar has made significant investments in sports infrastructure, such as building state-of-the-art fitness centers and extending bike routes, following the identification of weaknesses highlighted by its citizens through questionnaires (27, 28).

Nevertheless, one of the biggest deterrent to physical activity remains the harsh weather conditions (7, 8). Qatar is located in the Arabian Peninsula, characterized by an arid desert climate. Its temperatures range from below 5 °C in winter to over 45 °C in summer, averaging at around 27 °C for most of the year (29, 30). High humidity, especially in the summer, intensifies the heat and makes it challenging for people to perform extended physical activities outside (29). These extreme temperatures render outdoor activities as nearly impossible for most people, including locals who are generally more accustomed to the climate of the region. In order to support year-round physical activity, there has been a growing reliance on air-conditioned sports complexes and indoor fitness centers. In response, the government has implemented climate-adaptive measures, like chilled jogging routes, shaded outdoor areas, and nighttime athletic activities, to lessen the negative effects of intense heat and promote ongoing physical activity.

Despite these challenges, engaging in different forms of exercise remains crucial for managing T2DM. Physical activity has traditionally been categorized into two main forms: cardiovascular-focused (aerobic) and strength-focused (resistance/weight) exercises. Most literature on type 2 diabetics and physical activity has been looked at either aerobic or resistance training. Several studies have shown that aerobic (e.g., brisk walking and cycling) and resistance (e.g., weightlifting and resistance bands) exercise, both in isolation and more potently in combination, enhance skeletal muscle mass, improve insulin sensitivity, regulate blood pressure, and support weight loss (31–34). However, this binary model overlooks important aspects of physical health and overall well-being. Scientists have increasingly recognized the need to include two additional categories: balance and flexibility training (e.g., yoga and tai chi), which address physical skills that tend to decline with age, such as coordination, limb control, and stability (35, 36). Flexibility and balance training in individuals with T2DM, particularly among older adults and those with obesity, has been found to enhance joint flexibility, increase range of motion, and improve body balance, thereby reducing the risk of falls and promoting overall mobility (33). Thus, incorporating a combination of aerobic, resistance, balance, and flexibility training offers the most holistic approach to improving physical health, metabolic control, and overall well-being in individuals with T2DM.

Regarding the recommended amount of physical activity, it is generally accepted and standardized within the scientific community that all individuals, whether diabetic or not, should engage in at least 150 min of moderate-intensity aerobic activity per week, along with at least two days of muscle-strengthening exercises (33). Utilizing this guideline is particularly beneficial for those with T2DM, as regular physical activity enhances insulin sensitivity and reduces the risk of complications. Nevertheless, it is important to note that according to a 2022 WHO report in Qatar, physical inactivity remains a significant concern across age groups, with 49% of adult women and 33% of men aged 18 + not meeting recommended activity levels, and inactivity rates rise further among those aged 70+, affecting 64% of adult women and 48% of men (37). Among adolescents, 91% of females and 86% of males (ages 11–17) are physically inactive (37).

It is essential to contextualize the circumstances that the average type 2 diabetic experiences. Chronic fatigue, muscular weakness, neuropathy (damage to the nerves that causes pain or numbness, especially in the feet), and cardiovascular problems are some of the symptoms that a person with T2DM may experience that might make it difficult for them to exercise (38). They may find it especially difficult to engage in the required amounts of physical activity as a result of these reasons. To manage and control the condition, however, moderate to strenuous physical activity is highly recommended (33). Nevertheless, walking is one of the easiest ways to get regular physical activity. Remarkably, research has shown that walking for at least half an hour each day can lower the risk of developing T2DM by almost 50% by improving glycemic control and insulin sensitivity (39, 40). Additionally, among those who have already been diagnosed with the condition, regular walking has been linked to better overall health outcomes and decreased mortality rates (41).

Incorporating various types of exercise into everyday life is essential because of the substantial influence that physical activity has on the prevention and treatment of T2DM. Strategic adaptations, such climate-responsive infrastructure and customized exercise suggestions, can assist people with T2DM in maintaining an active lifestyle, even when environmental and physiological obstacles may still exist. Qatar can further its public health objectives and enhance the long-term health of its citizens by promoting a culture that values physical exercise through community involvement and policy. However, the long-term effectiveness of these interventions on physical activity levels and T2DM outcomes in Qatar remains insufficiently evaluated.

### 3.3 Policy and public health interventions in Qatar

The findings of this review have several important implications for public health policy and practice in Qatar. Prevention efforts must go beyond individual behavior modification and be integrated into national systems, community settings, and policy frameworks due to the high prevalence of T2DM and its strong correlation with modifiable lifestyle factors. This presents an opportunity to address T2DM risk in a manner that is both scalable and contextually relevant by including nutrition and physical activity interventions into the current public health infrastructure.

At the population level, dietary recommendations emphasizing low-glycemic, fiber-rich foods could be incorporated into national dietary guidelines, food subsidy programs, and public awareness campaigns to promote healthier eating patterns without compromising cultural food customs. Routine screening for lifestyle-related risk factors and structured lifestyle counseling could be incorporated into diabetes prevention and management pathways in primary care settings. School-based interventions such as nutrition education, better school food settings, and climate-adapted physical activity programs offered in indoor or shaded spaces offer a vital chance for early prevention.

Physical activity promotion strategies must also account for Qatar’s extreme climatic conditions by prioritizing indoor, climate-controlled, and time-adapted exercise opportunities in schools, workplaces, mosques, and community centers. Active lives may be further supported by workplace wellness programs and urban planning measures that promote incidental physical activity such as walking indoor areas and shaded infrastructure. In line with Qatar’s larger noncommunicable disease prevention plan, policies including front-of-pack food labeling, menu calorie transparency, and targeted fiscal measures could encourage healthier choices. Collectively, these multi-sectoral strategies underscore the importance of coordinated action across health, education, urban development, and policy domains in order to achieve sustainable reductions in T2DM risk.

### 3.4 Population heterogeneity and equity considerations

It is crucial to acknowledge that T2DM risk, lifestyle choices, and access to preventive resources are not equally distributed across the various sub-communities within Qatar, even though this review focuses on population-level strategies for T2DM prevention in Qatar. When developing and executing interventions, sex-, age-, and socioeconomic-specific characteristics that influence eating habits, opportunities for physical exercise, and involvement with health services should be taken into account.

Gendered norms related to physical activity participation, caregiving obligations, and access to recreational areas may be reflected in sex-based disparities in T2DM burden and health behaviors, especially for women (42). Age-related factors are especially important because preventive measures for children and adolescents such as school-based physical activity and nutrition programs differ significantly from those required for older populations or working-age individuals managing functional limits and co-morbidities. In the context of Qatar’s heterogeneous population composition, which comprises both Qatari nationals and expatriate workers with varying income levels, job circumstances, and access to healthcare facilities, socioeconomic disparities further impact T2DM risk and preventive chances. To guarantee that T2DM prevention techniques are equitable, culturally sensitive, and effective across demographic subgroups, it is crucial to address these aspects of heterogeneity.

### 3.5 Impact of the COVID-19 pandemic on lifestyle T2DM risk

The COVID-19 pandemic has had significant implications with regards to T2DM risk, lifestyle behaviors, and healthcare delivery in Qatar. Periods of lockdown and mobility restrictions were associated with reduced physical activity, increased sedentary behavior, and greater reliance on food delivery services; in conjunction, these factors potentially exacerbate metabolic risk factors (43). Disruptions to routine healthcare services and preventive screening may have further delayed the identification and management of individuals at high risk of T2DM (44). Consequently, the pandemic rapidly accelerated the adoption of telemedicine, remote lifestyle counseling, and home-based exercise programs, highlighting the potential and feasibility of digital and hybrid approaches to diabetes prevention and management (45). These experiences underscore the importance of developing prevention strategies that are resilient to environmental constraints and public health disruptions, while leveraging digital health solutions to support sustained lifestyle modification in Qatar.

## 4 Further research and considerations

Despite growing literature on T2DM in Qatar, significant gaps remain in the evidence base, particularly in longitudinal data, intervention effectiveness, and policy evaluation.

Although this review highlights evidence-based strategies for T2DM prevention and risk reduction, several priority areas warrant further investigation, particularly in the context of Qatar. Future research should focus on intervention studies evaluating the effectiveness of culturally tailored dietary programs that modify traditional eating patterns while preserving cultural acceptability. In parallel, climate-adapted physical activity interventions such as indoor, time-shifted, and community-based exercise models should be rigorously evaluated, especially among populations facing greater barriers to outdoor activity, including women, older adults, and individuals with obesity.

Further research is also needed to assess the real-world impact of policy-level interventions, including front-of-pack food labeling, menu calorie disclosure, and fiscal measures, on dietary behavior, physical activity, and downstream T2DM outcomes in Qatar. The evidence base would be strengthened by longitudinal studies examining the influence of screen time, sedentary behaviors, and digital food delivery platforms on metabolic health. Finally, studies exploring the role of digital health and telemedicine solutions such as home-based exercise programs, remote lifestyle counseling, and mobile health interventions may help identify scalable approaches to diabetes prevention and management that are resilient to environmental constraints and future public health disruptions.

Given the multifaceted nature of lifestyle influences on T2DM, a comprehensive analytical approach focusing on nutrition and physical activity as primary intervention domains is also warranted. These two lifestyle components represent the most modifiable and impactful factors for T2DM prevention and management, offering the greatest potential for population-level health improvements. To address T2DM in Qatar through nutritional changes, several modifications to the traditional diet can be made without compromising its cultural significance. Many Qatari dishes are rice based and rely on white rice, which has a high glycemic index meaning that it can quickly raise blood glucose levels (15). Replacing white rice with other alternatives such as quinoa or brown rice can help improve blood sugar regulation as they are whole grain alternatives with lower glycemic index. Similarly, incorporating more vegetables into dishes or adding legumes such as chickpeas or lentils to meals increases fiber content, which is essential for stabilizing blood glucose levels (46). Food preparation methods also play a major role as deep fried dishes are higher in calories and contain trans fats which are associated with an increased risk of developing T2DM (11). Opting for alternative food preparation options such as air-frying can reduce the oil content and overall calorie density of meals. Small adjustments like these can have a lasting impact on managing T2DM overtime. Furthermore, promoting gut-friendly foods including cultured yogurt, onions, and garlic could help control insulin (47).

Effective nutritional interventions must address both macronutrient composition and eating behavior patterns since most Qataris consume their main meals at home rather than restaurants, creating change within households is essential. Engaging prominent individuals in the local culinary scene to promote healthier versions of traditional recipes can encourage families to adopt better eating habits. Furthermore, the Ministry of Trade and Industry could implement regulations mandating restaurants to include calorie and nutritional information in their menus. This initiative would help increase awareness of the nutritional value of meals and enable individuals to make better informed choices when dining out. While dietary patterns significantly influence T2DM risks, physical activity plays an equally crucial role in both prevention and management.

Moreover, while severe weather often makes outdoor exercise difficult, the Aspetar-led National Physical Activity Guidelines provide evidence-based approaches to prevent chronic diseases. Physical activity interventions must be tailored to Qatar’s unique environmental and cultural context. This includes developing climate-appropriate exercise programs, creating supportive environments, integrating physical activity into daily routines, and addressing cultural barriers to exercise participation particularly benefiting older adults and women who may face additional barriers to regular exercise.

The promotion of gut health has also been identified as an effective strategy in T2DM management. Diets that include prebiotic foods such as garlic and onions, probiotics from fermented foods like yogurt and kefir, and a variety of high-fiber foods such as vegetables and whole grains have been shown to support a healthy gut microbiome (47). A diverse and balanced microbiome is associated with reduced systemic inflammation, improved glucose metabolism, and a lower risk of diabetes (48). These dietary elements are often incorporated into daily meals, such as pairing fermented yogurt with rice or including a side salad alongside main dishes.

In order to gather thorough data that will guide future public health policy, it is imperative that screen time, eating patterns, and levels of physical activity be continuously monitored. By combining these coordinated actions, Qatar may aggressively combat the growing prevalence of diabetes, promote long-term health sustainability, and preserve cultural traditions.

## 5 Conclusion

In Qatar, type 2 diabetes mellitus represents a complex and escalating public health challenge driven by the interaction of lifestyle behaviors, environmental constraints, and broader policy contexts. Addressing gaps in existing regional literature that often considers nutrition, physical activity, and policy interventions in isolation, this review synthesizes current evidence to advance a Qatar-specific, climate-adaptive, and culturally aligned framework for T2DM prevention and risk reduction. By explicitly situating lifestyle determinants within Qatar’s extreme climatic conditions, sociocultural dietary practices, and national public health landscape, this review contributes a contextualized perspective that is directly relevant to local prevention efforts.

The findings underscore the need for multi-sectoral strategies that integrate nutrition reform, climate-adapted physical activity promotion, and supportive policy measures within existing health, education, and urban systems. For clinical practice, this framework highlights opportunities to strengthen lifestyle counseling and early risk identification within routine care. For public health practice, it emphasizes the importance of community-based, culturally sensitive, and equity-oriented interventions. At the policy level, coordinated action across sectors including food systems, urban planning, and health promotion is essential to achieve sustainable reductions in T2DM risk. While implementation challenges remain, including behavioral adherence, resource allocation, and population heterogeneity, the integrated framework proposed in this review provides a foundation for future research, policy development, and intervention design. Although focused on Qatar, the principles outlined may also inform T2DM prevention strategies in other GCC and hot-climate, rapidly urbanizing settings facing similar challenges.
